# Proposing a surgical algorithm for graduated orbital decompression in patients with Graves’ orbitopathy

**DOI:** 10.1007/s00405-021-07003-0

**Published:** 2021-07-21

**Authors:** Kerstin Stähr, Anke Daser, Michael Oeverhaus, Timon Hussain, Stephan Lang, Anja Eckstein, Stefan Mattheis

**Affiliations:** 1 Department of Otorhinolaryngology, Head and Neck Surgery, University Hospital Essen, Hufelandstr. 55, 45147 Essen, Germany; 2 Department of Ophthalmology, University Hospital Essen, Essen, Germany

**Keywords:** Graves’ orbitopathy, Orbital decompression, Diplopia, Exophthalmos, Surgical outcome

## Abstract

**Purpose:**

To determine the outcome after orbital decompression using a graduated technique, adapting the surgical technique according to individual patients’ disease characteristics.

**Methods:**

We retrospectively examined the postoperative outcome in patients treated with a graduated balanced orbital decompression regarding reduction of proptosis, new onset diplopia and improvement in visual function. 542 patients (1018 orbits) were treated between 2012 and 2020 and included in the study. Clinical examinations including visual acuity, exophthalmometry (Hertel) and orthoptic evaluation were performed preoperatively and at minimum 6 weeks postoperatively. Mean follow-up was 22.9 weeks.

**Results:**

Mean proptosis values have significantly decreased after surgery (*p* < 0.01). In 83.3% of the patients Hertel measurement normalized (≤ 18 mm) after surgery, New onset diplopia within 20° of primary position occurred in 33.0% of patients, of whom 16.0% had preoperative double vision in secondary gaze. Patients suffering from dysthyroid optic neuropathy (DON) had a significant increase in visual acuity (*p* < 0.01).

**Conclusion:**

We demonstrated that individually adapted graduated orbital decompression successfully improves key disease parameters of Graves’ orbitopathy with low morbidity.

## Introduction

Graves’ orbitopathy (GO) is the most common reason for orbital decompression surgery and occurs in about 50% of patients suffering from Graves’ disease. Clinical symptoms include proptosis due to an increased volume of orbital tissue, lid retraction, inflammatory soft tissue reaction and muscle fibrosis leading to impaired eye motility which can cause diplopia, [1, 2]. An anti-inflammatory immunomodulatory therapy, e.g., i.v. steroids ± immune suppression or orbital irradiation, is applied during the active progressive phase [3]. If conservative therapy fails, surgical treatment may become necessary. Beside exophthalmos and incriminating inflammation, about 3–5% of patients develop a sight-threatening disease stage caused by corneal breakdown or dysthyroid optic neuropathy (DON), which require immediate surgical orbital decompression if high doses of i.v. steroids fail [3, 4]).

Today, different approaches for orbital decompression are at the surgeon’s disposal. Many of them are based on minimally invasive techniques and include a removal of the medial, lateral and inferior wall or a combination of these [5–8]. While lateral decompression alone has the lowest rate of new onset diplopia (NOD) (3–8%), it only results in a relatively small decrease in the Hertel Index of about 3–4 mm [9–12]. Medial decompression has the highest rates of NOD (35–40%), but with greater exophthalmos reduction than lateral decompression [13, 14]. We use a graduated approach in which the surgical technique is adapted to the individual needs of the patient. Our most commonly applied technique is based on the “balanced orbital decompression”, as described by Leone et al. [15]. With moderate rates of NOD (7–35%), this approach allows for the greatest variability of exophthalmos reduction to be achieved [16–20]. If the inferior medial strut and the anterior medial wall as well as the orbital floor are preserved, the exophthalmos regression as well as the strabismus rate is lower [21, 22]. By removing these structures, even severe exophthalmos can be corrected. In this way, preoperative asymmetrical findings can be compensated.

We retrospectively examined the postoperative outcome after decompression in terms of reduction of proptosis, new onset diplopia and visual improvement in case of DON in 542 patients (1018 orbits) after graduated balanced orbital decompression. Special attention was paid to the postoperative symmetry of the eyes, as many patients have a side difference of the exophthalmos preoperatively.

## Patients and methods

This study was based on a retrospective chart review. Medical records of patients treated in an orbital center of a tertiary care University Hospital for GO and undergoing graduated balanced orbital decompression between 2012 and 2020 were evaluated. The study was performed according to the Declaration of Helsinki and was approved by the local ethics committee. Only patients who met the following criteria were included: preoperative CT scan, complete ophthalmological examination pre- and postoperatively, ≥ 18 years of age. Informed consent was not necessary because of the retrospective nature of this analysis. Clinical assessments were performed immediately before surgery, and at the earliest 6 weeks postoperatively. All patients underwent routine ophthalmological examinations, including visual acuity, exophthalmometry, orthoptic evaluation, slit lamp and funduscopy. In addition, the age, duration of disease, gender and smoking status of the patients were recorded. To evaluate the surgical outcome regarding reduction of proptosis, the number of patients with a postoperative Hertel measurement within normal values (Hertel values 18-13 mm) was assessed. These limits were set according to the findings of Migliori et al. and our own previously unpublished clinical observations [23]. In addition, the difference between the Hertel measurements of both sides was calculated to estimate the achieved symmetry of the eyes. A difference ≤ 1 mm was considered a satisfactory outcome. The postoperative outcome in case of DON was evaluated based on visual acuity. A difference of more than one line difference in the Snellen visual acuity measurement was defined as a significant change. Pre- and postoperative diplopia of the subjects was determined by measurement of squint angle in primary and reading position using the prism-cover-test, assessment of the field of binocular single vision (BSV). In addition monocular excursions were measured with Kestenbaum glases. To investigate a possible influence of the amount of proptosis reduction on the rate of new onset diplopia (NOD), the percentage of patients with NOD at low (< 4 mm), moderate (4–6 mm) and high (> 6 mm) exophthalmos reduction was assessed.

### Surgical procedure

**Fig. 1:**
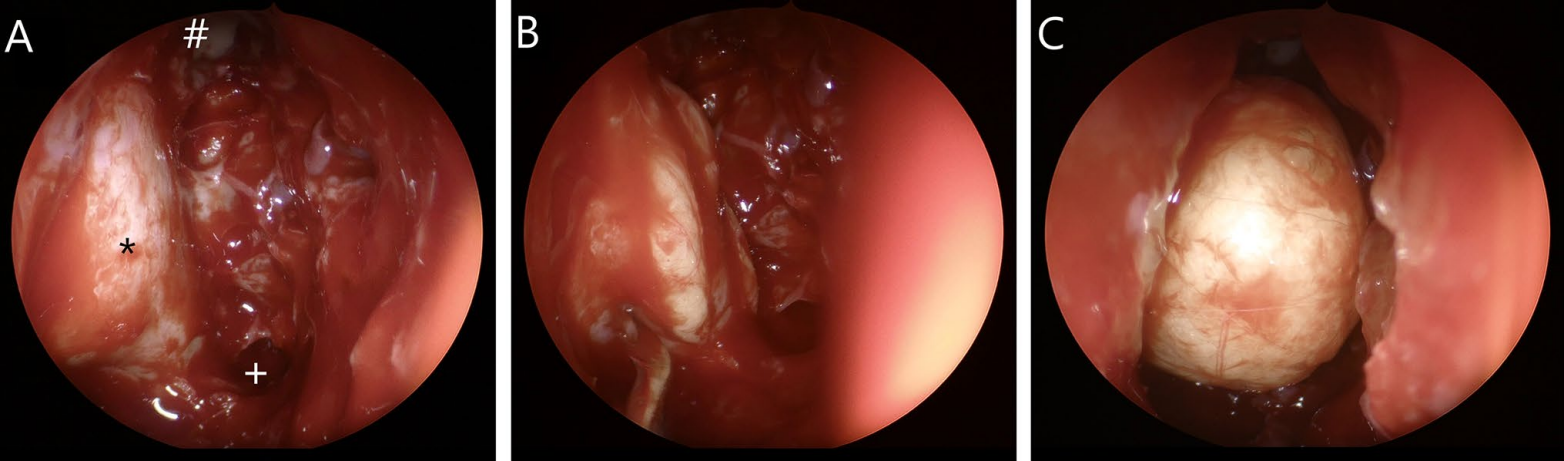
**A** Endoscopic view after resection of lamina parpyracea, #: frontal sinus, *: periorbit, +: sphenoid sinus, **B** resection of the periorbit using a sickle knife, **C** view after medial orbital decompression

**Fig. 2:**
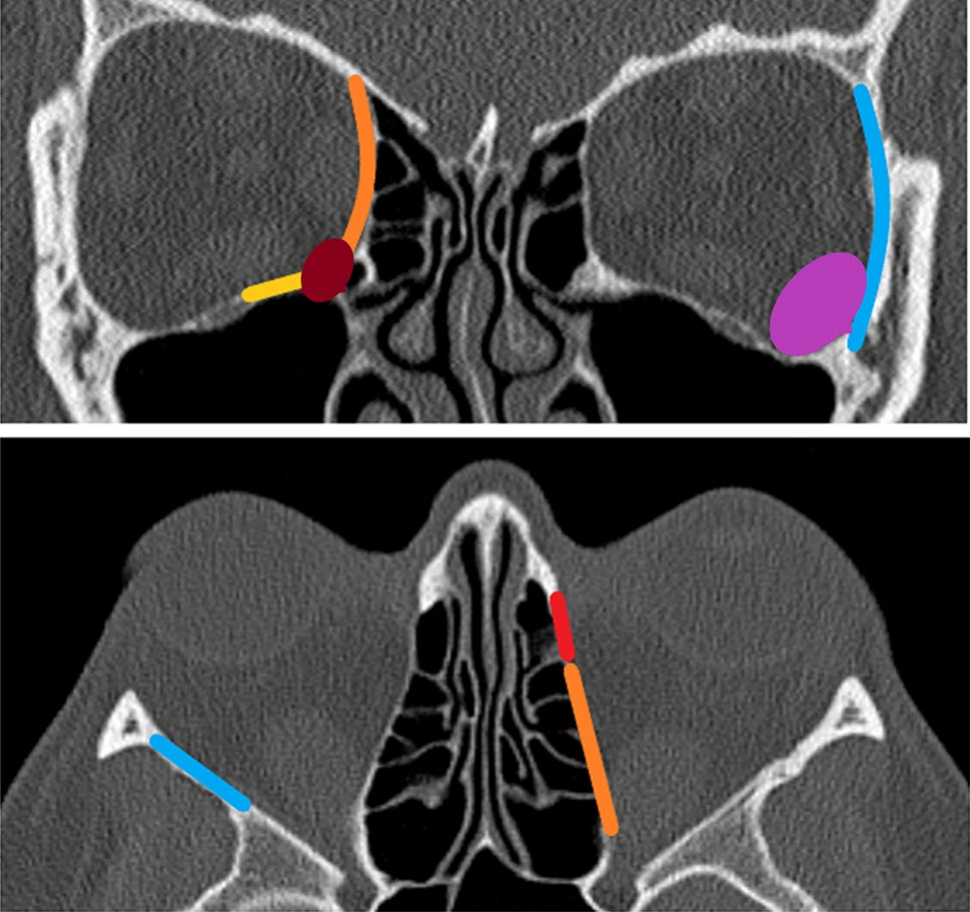
Graduated orbital decompression: lateral decompression including decompression of the deep lateral wall (blue), resection of 1.5–3 ml orbital tissue (purple); medial orbital decompression: resection of lamina parpyracea (red) with extension anteriorly (purple), resection if the inferior medial strut (dark red) and resection of the orbital floor up to the infraorbital nerve (orange)

All surgeries were performed under general anesthesia. Xylometazolin-soaked swabs were temporarily inserted for decongestion of the nasal mucosa. After removal of the uncinate process, the anterior and posterior ethmoid was resected exposing the skull base. The natural ostia of the maxillary sinus, frontal sinus and sphenoidal sinus were opened wide. The lamina papyracea was fractured and removed. In severe cases, additionally parts of the orbital floor were resected medial to the infraorbital nerve. In minor cases, the anterior part of the lamina papyracea was preserved to reduce the amount of dislocation of the medial rectus muscle and postoperative diplopia. Especially in case of DON, an adequate decompression of the optical nerve located in the orbital apex was ensured. The periorbita was slit from posterior to anterior and ablated. By gentle pressure applied on the bulb, led to the orbital contents prolapsing into the ethmoid cavity (Fig. 1). For resection of the lateral wall, a 10-mm incision was made laterally of the lateral cantus. The lateral orbital wall between the frontozygomatic suture and the zygomatic arch was removed using piezosurgery. Remaining edges of the deep lateral wall were smoothed with a high-speed burr. After excision of the periorbita, the orbital contents prolapsed into the newly created cavity. Additionally, up to 3 ml of orbital fat could be extracted, if necessary. To restore the contour of the orbit, the lateral orbital rim was replanted with microplates. All surgeries were performed by two ENT surgeons (S.M., K.S.). According to our algorithm, decompression took place as follows: if a minor reduction of exophthalmos (< 4 mm) was required, medial and lateral orbital walls were resected. The anterior parts of the inferior medial strut and the medial wall were preserved to reduce incidence of NOD. To achieve a moderate reduction of exophthalmos (4–6 mm) the resection of the medial wall was extended anteriorly and included the inferior medial strut. Additionally the deep lateral wall was reduced as far as possible and about 1.5–3 ml of orbital fat was extracted. If a high amount of reduction (> 6 mm) was needed, the medial part of the orbital floor up to the inferior orbital nerve was resected. In extreme cases of exophthalmos, replantation of the lateral orbital rim was not performed. The scheme shown here gives an approximate indication of the structures to be resected, but must be partially adapted by the experienced surgeon. This is due to two factors: the individual rigidity of the orbital tissue determines the soft tissue prolapse and thus the decrease in exophthalmos. Particularly in patients who have severely enlarged extraocular eye muscles, an insufficient decrease in proptosis can occasionally be observed. The individual anatomy of the orbit also determines the reduction of exophthalmos. The larger the area of the medial and lateral orbital wall, the greater the possible prolapse of the orbital tissue [24] (Fig. 2).

### Statistical analysis

IBM SPSS Statistics 26.0 (Chicago, IL, USA) was used for statistical analysis. For metric data mean, range and standard deviation (SD ±) were calculated. Data analysis to compare pre- and postoperative measurements was performed using the Wilcoxon signed-rank test. Statistical significance was defined as *p* < 0.05. Pearson’s Chi-squared test was used to determine possible statistic differences in NOD in patients with low, moderate and high exophthalmos reduction.

## Results

### Patients

Of all patients receiving orbital decompression due to GO between August 2011 and November 2020 542 (1018 orbits) matched all inclusion criteria. Their postoperative examination took place after an average of 22.9 months (range 6–82 weeks). Female to male ratio was 5:1. Dysthyroid optical neuropathy (DON) was present in 365 orbits. Further details on patients’ characteristics are provided in Table 1. Adverse events were postoperative temporary edema (*n* = 190 patients, 35%) which improved already during the stay in the hospital, intermittent infraorbital hypesthesia (*n* = 22 patients, 4%) and intraoperative CSF leaks (*n* = 7 patients, 1%). All CSF leaks could be closed directly intraoperatively using fascia lata and TachoSil^®^.

**Table 1:** Patients’ demographics, smoking habits and preoperative characteristics

Variable	N64%
Age at surgery in years (range)	51.6 (18–86)
Gender (male/female)	90/452
Duration of EO at surgery in years (range)	3.6 (0.1–23.8)
Smoker (*n*)	302 (55.6%)
Orbital radiotherapy (*n*)	179 (32.9%)
Steroid therapy (*n*)	453 (83.7%)
Preoperative diplopia	347 (64%)
In primary position	185 (34%)

### Reduction of proptosis and symmetry of the eyes

**Fig. 3:**
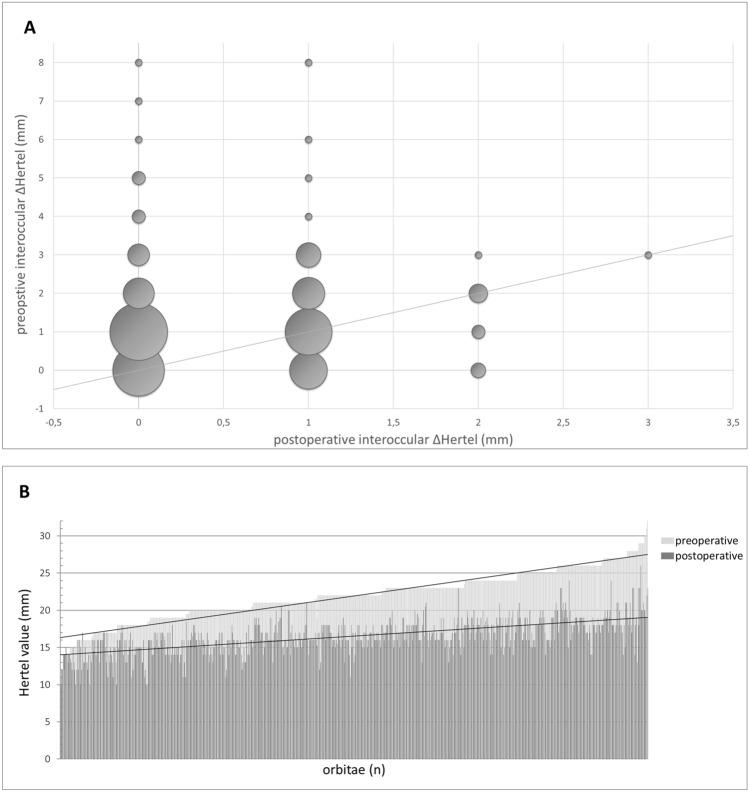
**A** Symmetry of the eyes. **B** Comparison of pre- and postoperative Hertel measurements, both trendlines illuminating the difference of reduction of proptosis in patients with high preoperative Hertel measurement compared to those with lower values

Mean preoperative Hertel measurement was 22.1 mm (range 15–32) vs. 16.7 mm (range 10–26), postoperatively. Exophthalmos was significantly (*p* < 0.01) reduced by 5.6 mm (SD 2.3), 78.9% (*n* = 427) of all patients exhibited postoperative Hertel measurements within the normal range (13–18 mm), and 91.8% (*n* = 331) of all patients reached values < 20 mm. 23 patients had postoperative Hertel measurements below 14 mm (4.2%). Figure 3 shows that a high reduction of proptosis was achieved in patients with a high preoperative Hertel measurements, while a significantly lower reduction was achieved in patients with low degrees of preoperative proptosis. There was no significant difference in preoperative Hertel measurements in patients without (22.2 mm) and with DON (21.8 mm) (*p* = 0.057). A symmetry (∆ Hertel ≤ 1 mm) of the postoperative Hertel measurements of both eyes was established in 93.1% (*n* = 494) of the patients (Fig. 3).

### Outcome in patients suffering from DON

**Fig. 4:**
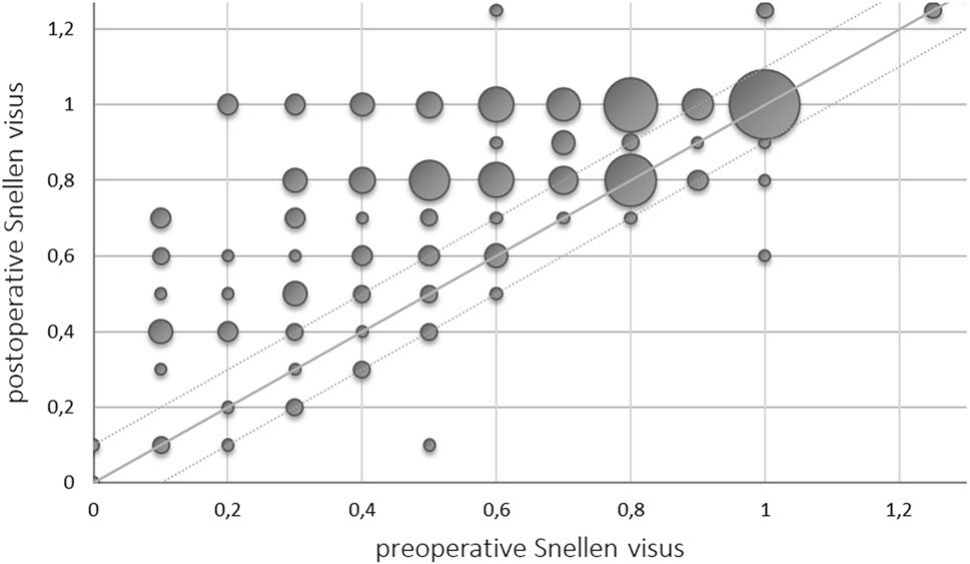
Changes in visual acuity data points above the angel bisector indicate improved vision. Differences of ± 0.1 were considered as measurement inaccuracy

The visual acuity in patients suffering from DON increased significantly (*p* < 0.001) from 0.6 (range no light perception—1.25) preoperatively to 0.8 (range no light perception—1.25) postoperatively. The eyesight after surgery decreased in four eyes (1.1%, three patients). In addition, two of these patients were affected by severe glaucoma and one diabetic patient suffered from a heavy reactivation of the GO 2 weeks after surgery. Visual acuity increased in 171 eyes (46.8%) and stabilized in 190 eyes (52%) of which 75 (20.1%) had a preoperative visual acuity ≥ 0.8. Figure 4 shows the ratio of preoperative to postoperative visual acuity.

### New onset diplopia

**Fig. 5:**
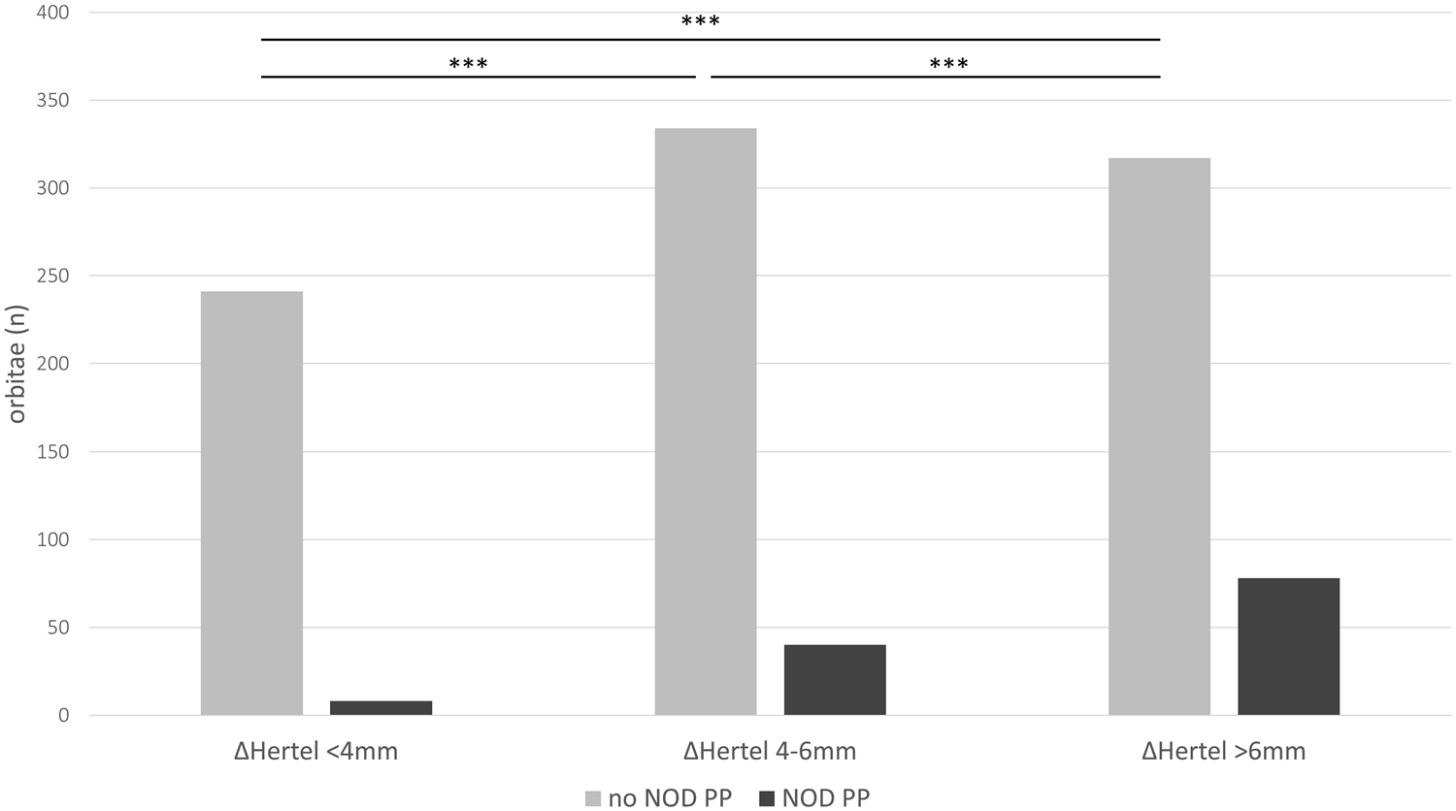
Incidence of NOD depending on exophthalmos reduction

Preoperatively, 347 (64%) of patients suffered from double vision, of which 185 (34%) had diplopia in primary position. Postoperatively, 446 (77%) subjects had double vision, including 341 (63%) cases with diplopia in primary position. New onset of diplopia in primary position in patients with no preoperative diplopia occurred in 64 individuals (33% of all patients with no preoperative diplopia). In Orbits with low exophthalmos reduction (< 4 mm, *n* = 253 orbits) NOD occurred in 8 cases, in orbits with moderate exophthalmos reduction (4-6 mm, *n* = 374 orbits) in 40 cases and in orbits with high exophthalmos reduction (> 6 mm, *n* = 391) in 78 cases. The frequency of NOD was significantly different between the groups (*p* < 0.001) (Fig. 5).

## Discussion

In this retrospective single-center study, a large cohort of patients suffering from GO was examined. Surgical goals in orbital decompression include optical nerve decompression, proptosis reduction, reaching a normal Hertel value, achieving symmetry of the eyes and different combinations of these. Our data suggest that addressing these different topics with an adapted technique of balanced orbital decompression leads to the best results. Patients’ age, gender distribution, smoking status and the type of pre-treatment were on par with comparable patient collectives.

### Reduction of proptosis and symmetry of the eyes

With an average decrease of 5 mm, our degree of reduction is within the range of levels attained by other groups (4.1–5.7 mm) [17–19, 25–27]. However, since our surgical approach is a graduated approach, this parameter alone holds limited significance. While a maximum reduction may be desired in certain cases, this may not apply in patients with moderate exophthalmos. In those cases, optical decompression or symmetry might be more important. In our opinion, the rate of patients who reached normal Hertel-values provides a more accurate indication of the success of the surgical technique. This could mean a proptosis reduction of 2 mm in minor cases as well as 12 mm in severe cases. We consider a post-therapeutic normalization of Hertel measuremet (13–18 mm) values in 83.3% of cases to be a satisfying outcome. Tailored surgery provides the opportunity to deal both with minor and severe cases leading to a comparable outcome in both groups. Hereby, a symmetrical postoperative outcome was achieved in 93.1% of patients, reinforcing the notion of a successful surgical strategy. In 23 patients, the postoperative Hertel value was below the normal values, so that the decline was slightly higher than aimed for. In these cases, lateral decompression alone, in the absence of DON, may be more beneficial.

### Outcome in patients suffering from DON

Patients suffering from DON showed a significant improvement in visual acuity. In 99% of the subjects, visual acuity was stabilized, or improved postoperatively. Park et al. recently showed that orbital decompression leads to a decrease in peripapillary thickness, which is associated with an improvement in visual acuity [28]. This is in line with the findings of other authors who showed an improvement in visual acuity in patients with DON after orbital decompression [17, 26, 29, 30]. However in three of our subjects we did not achieve an improvement in visual acuity after decompression. Chart review suggests that one of these specific patients suffered from severe, and rapidly progressing disease and first presented at our institution at an advanced disease stage. The other two patients also suffered from glaucoma, so we assume that the lack of improvement in visual acuity is due to this. Of the patients who had stabilization of visual acuity, 75 already had a preoperative visus of ≥ 0.8, so further improvement was not necessarily expected. Furthermore, according to our clinical experience, the visual acuity still improves within the first year, which was partly outside the follow-up. A further study could be useful to clarify this.

### New onset diplopia

The definition of rate of new-onset diplopia (NOD) varies. Pariseans et al. consider the NOD in primary position as most relevant, which we have followed [17]. Our rate of 33% NOD is in line with those achieved by other groups using a similar surgical technique [19]. According to Rocchi et al., preoperative diplopia in secondary gaze is a strong risk factor for NOD, which our analyses confirm [18]. Different authors described a relatively high rate of NOD after resection of the medial orbital wall (35–40%)[13, 14], that is up to 50% after resection of the medial and inferior wall [31]. If the lateral wall alone was resected, NOD occurred in only 3–8% [9–12]. Using techniques that combined the resection of the medial and the lateral wall the reported rate of NOD is about 7–35% [16–20]. Accordingly, the rate of NOD in our collective was lowest in patients with low requirement of exophthalmos reduction after resection of the lateral wall and the posterior part of the medial wall. After additional removal of the anterior part of the medial wall and the inferior medial strut we observed an increase of NOD, corresponding to the findings of Bleier et al. and Wright et al. [21, 22]. Our highest rate of NOD was detected after resection of the medial, the medial inferior and the lateral wall. However, this technique allowed the highest reduction of exophthalmos and normalisation of the Hertel values even in the most severe cases. We conclude, that avoidance of NOD should not be the only goal after orbital decompression, but achieving normal Hertel-Values and symmetry of the eyes is as important as well.

## Conclusion

A graduated approach for surgical balanced orbital compression addresses patients’ specific requirements while achieving satisfying postoperative results. A customized reduction of exophthalmos based on the preoperative state is possible, as is a sufficient decompression of the optic nerve in neuropathy.
